# Therapeutic Effects of Melatonin on Ocular Diseases: Knowledge Map and Perspective

**DOI:** 10.3389/fphar.2021.721869

**Published:** 2021-11-02

**Authors:** Haozhe Yu, Qicong Wang, Wenyu Wu, Weizhen Zeng, Yun Feng

**Affiliations:** ^1^ Department of Ophthalmology, Peking University Third Hospital, Beijing, China; ^2^ Institute of Medical Technology, Peking University Health Science Center, Beijing, China; ^3^ Department of Chinese Medicine of Taiwan, Hong Kong and Macao, Beijing University of Chinese Medicine, Beijing, China

**Keywords:** melatonin, circadian rhythms, ocular diseases, therapeutics, pharmacology

## Abstract

Melatonin plays a critical role in the pathophysiological process including circadian rhythm, apoptosis, and oxidative stress. It can be synthesized in ocular tissues, and its receptors are also found in the eye, triggering more investigations concentrated on the role of melatonin in the eye. In the past decades, the protective and therapeutic potentials of melatonin for ocular diseases have been widely revealed in animal models. Herein, we construct a knowledge map of melatonin in treating ocular diseases through bibliometric analysis and review its current understanding and clinical evidence. The overall field could be divided into twelve topics through keywords co-occurrence analysis, in which the glaucoma, myopia, and retinal diseases were of greatest research interests according to the keywords burst detection. The existing clinical trials of melatonin in ocular diseases mainly focused on the glaucoma, and more research should be promoted, especially for various diseases and drug administration. We also discuss its bioavailability and further research topics including developing melatonin sensors for personalized medication, acting as stem cell therapy assistant drug, and consuming food-derived melatonin for facilitating its clinical transformation.

## Introduction

Melatonin is a pleiotropic hormone synthesized from serotonin, which is mainly secreted by the pineal gland controlled by the hypothalamic suprachiasmatic nucleus (SCN) (Gillette and McArthur, 1995). The secretion of melatonin presents the character of increasing at night and decreasing during the day, indicating its role in regulating circadian rhythms (Fedele et al., 2018). Besides, the melatonin also demonstrates superior properties in antioxidant, immunomodulation, and neuroprotection (Mayo and Sainz, 2020; Moradkhani et al., 2020; Ramos et al., 2020). The function realization of melatonin depends on the receptor-independent or -mediated processes, and MT1 and MT2 are the main receptors, both of which belong to G-protein–coupled receptors and are widely distributed in various tissues (Singh et al., 2017; Legros et al., 2020). MT3 is the low-affinity receptor for melatonin, which is considered an enzyme with different characteristics, compared with MT1 and MT2 including kinetics in the ligand association/dissociation and pharmacological profile (Paul et al., 1999; Nosjean et al., 2001).

Given that the photoreceptive retinal ganglion cell is the important zeitgeber of the SCN and the circadian rhythms can be influenced under suffering ocular diseases, the relationship between melatonin and eye has attracted much attention (Turner et al., 2010; Andrews et al., 2019). Several studies have reported that the melatonin could be produced in various ocular tissues following the circadian rhythms including the lachrymal gland, retina, crystalline lens, iris, and ciliary body (Mhatre et al., 1988; Faillace et al., 1995; Alkozi et al., 2017b; Alkozi et al., 2017c). The melatonin receptors were also widely detected in the eye, such as sclera, cornea, choroid, and retina (Savaskan et al., 2002; Wiechmann and Rada, 2003; Summers Rada and Wiechmann, 2006). Much research revealed the correlation between melatonin with various ocular conditions, especially for glaucoma, inflammatory, and age-related diseases, as well as explored the therapy methods based on melatonin (Aranda et al., 2017; Crooke et al., 2017; Alkozi et al., 2020). This perspective will incorporate the existing studies based on the knowledge map and clinical trials of melatonin in ocular diseases, which aims to provide novel insights into promoting the melatonin from the bench to bedside from the point of view of enhancing the bioavailability and future research direction based on pharmacological issues.

### Melatonin in Ocular Diseases

**FIGURE 1:**
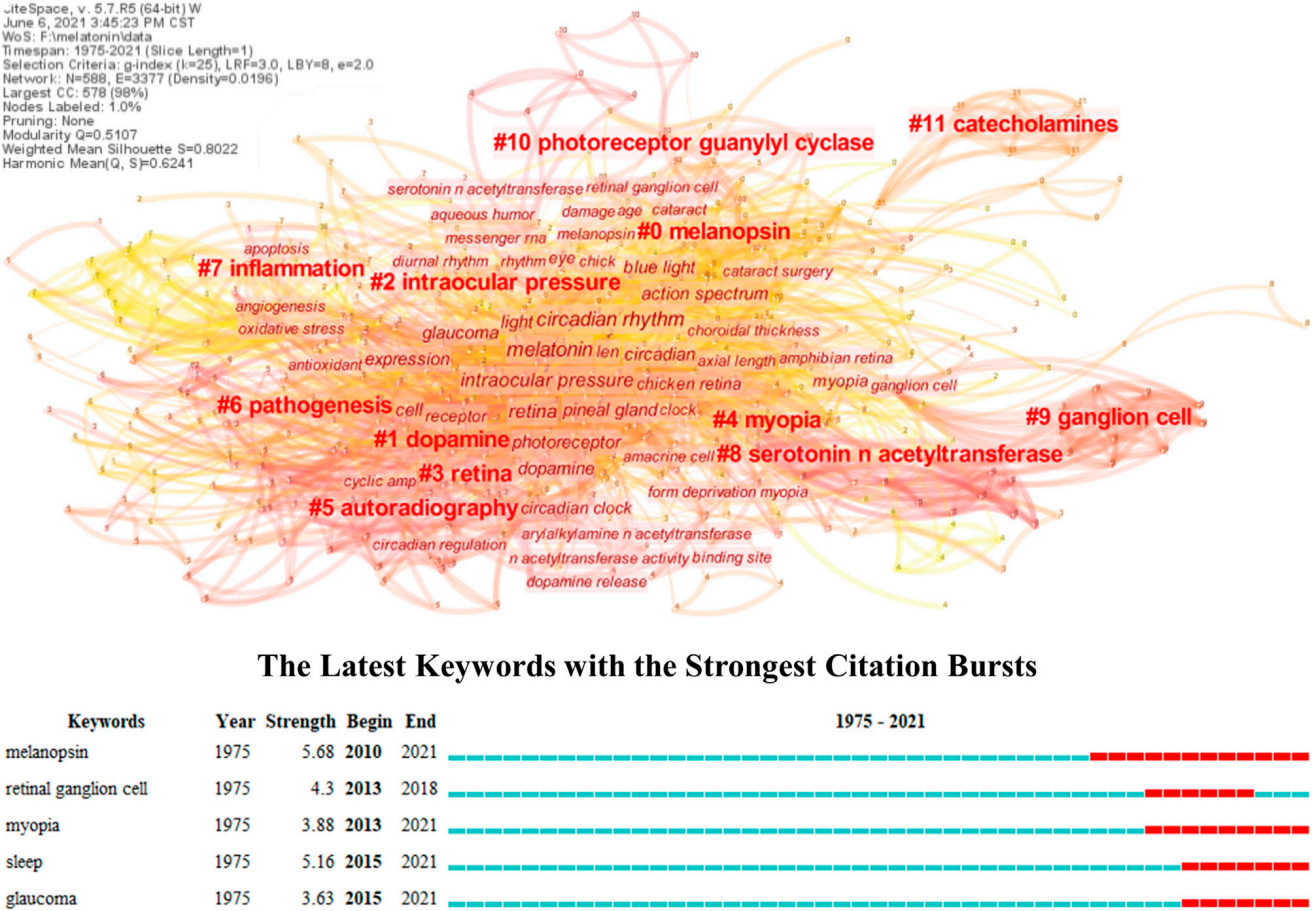
Knowledge map of melatonin treating ocular diseases based on keywords co-occurrence and keywords burst detection.

A knowledge map based on bibliometric analysis can present the overall research topics and trends compared with the topical review, which provides an in-depth insight into its frontiers and hotspots in this field (Deng et al., 2020; Valera-Gran et al., 2020). However, the role of melatonin in ocular diseases has not yet been analyzed through this method as far as we know. Therefore, its knowledge map was constructed in this research through keyword co-occurrences, and the keyword burst was also conducted to explore its trends. As shown in Figure 1, all the studies of melatonin in ocular diseases could be divided into twelve different clusters, and their connections and average year appeared were reflected by the thickness (closely in thick) and color (newly in yellow) of lines, respectively. #0 melanopsin is an opsin located in the retina and crystalline lens epithelial cells, which plays an important role in visual functions like detection and color, and non-visual functions like regulating pupil size and melatonin secretion (Hannibal et al., 2017; Prayag et al., 2019b; Spitschan, 2019). The melanopsin is sensitive to 480 nm blue light and leads to the low expression of the melatonin synthesis enzyme AANAT, which can be used to understand the mechanism of sleep disturbances and depression in patients with cataracts and retinal diseases (Feigl and Zele, 2014; Shenshen et al., 2016; Alkozi et al., 2017c; Münch et al., 2017). The influences of melanopsin in ocular diseases have been proven, and several research studies developed novel therapy methods in regulating the melatonin content in the eye including wearing yellow filter for controlling intraocular pressure (IOP) (Lledó et al., 2019; Zheng et al., 2020). #1 Dopamine and melatonin together organize the retinal circadian rhythmicity through dopamine D-4 and MT1 receptors, respectively; however, the former is mainly synthesized during the day indicating the different phase relationship with melatonin (Adachi et al., 1998; Bartell et al., 2007; Kunst et al., 2015; Goel and Mangel, 2021). They take opposing roles in regulating physiological functions of the eye, and the dopamine can reduce the expression of AANAT, resulting in the limitation of melatonin synthesis (Zawilska et al., 2004; Lorenc-Duda et al., 2009; Lavoie et al., 2010; Lavoie et al., 2013). It has been reported that the dopamine D-3 receptor could form heteromers with the MT1 or MT2 receptor and presented a negative correlation with intraocular hypertension, which might impact the occurrence of glaucoma (Reyes-Resina et al., 2020). Besides, the dopamine has been well-studied in myopia; however, its potential relevance with melatonin needs to be further explored (Wang et al., 2021c; Landis et al., 2021). #5 autoradiography, #8 serotonin *N*-acetyltransferases, #9 ganglion cell, #10 photoreceptor guanylyl cyclase, and #11 catecholamines represent the fundamental research of melatonin including its receptor distribution, synthesis, responses to light, regulation, and photoreceptor degeneration (Falcon et al., 1991; Mazurais et al., 1999; Benyassi et al., 2000; Sato et al., 2018; Prayag et al., 2019a). Such topics are not directly connected to the ocular diseases; therefore, they are in the marginal positions of the knowledge map.

The #2 intraocular pressure, #3 retina, #4 myopia, #6 pathogenesis, and #7 inflammation reflect the main research topics of melatonin in ocular diseases. Combining with keywords burst detection, it can be seen that the myopia, glaucoma, and retinal diseases, especially age-related diseases, are the most concerned diseases in the clinical practice, while sleep is the major intervention. Several research studies have reviewed the influence of melatonin on IOP and glaucoma, especially emphasizing the role of circadian rhythms (Alkozi et al., 2020; Ciulla et al., 2020; Martinez-Aguila et al., 2021). Exogenous application of melatonin or its analog performs well for controlling IOP in both animal models and clinical trials, and its neuroprotective effect can further prevent retinal injury under intraocular hypertension (Carracedo-Rodríguez et al., 2020; Gubin et al., 2021). Melatonin can act as antioxidant, anti-inflammatory, and immunomodulation agents besides neuroprotection, which demonstrated superior therapy effect in retinal diseases, such as age-related macular degeneration (AMD) and diabetic retinopathy (DR), and immunologic ocular diseases like uveitis (Sande et al., 2014; Chesnokova et al., 2016; Diéguez et al., 2020; Ferreira de Melo et al., 2020). Furthermore, melatonin also regulates the secretion of vascular endothelial growth factor (VEGF) in the retina, and it promotes physiological secretion for protecting the retina from oxidative stress, while reduces pathological secretion for inhibiting neovascularization (Klettner et al., 2021). It has been reported that the refractive error, optical axial length, and power demonstrated diurnal variation, and the myopes presented higher melatonin concentrations in serum and salivary, while lower in urine than non-myopes (Campbell et al., 2012; Kearney et al., 2017; Flanagan et al., 2020; Chakraborty et al., 2021). Compared with emmetropes, the myopes generally have much evening-type diurnal preference with approximately 1 h phase-delay but no significant difference in outdoor light exposure, and such properties are expected to further understand the mechanism and effect of outdoor time and light environments in myopia control (Burfield et al., 2019; Wang et al., 2021b). Moreover, melatonin has been also widely used to reveal the pathogenesis and provide possible therapy methods for many other ocular diseases, including cataract based on the oxidative stress (Kiliç et al., 2008; Ohanness et al., 2009). Nonetheless, the exact role of melatonin in ocular diseases needs to be further explored, especially for the relationship between its biological activity and circadian rhythm regulation. Certain research proposed the secretion and signaling of melatonin were under control of the circadian rhythm, which could further influence its pleiotropy (Tosini and Menaker, 1996; Hardeland, 2019). However, it has been proven that the melatonin in myopia seemed independent of the circadian rhythm, and the research about how the circadian rhythm impacted the molecular mechanism of ocular disease occurrence is still lacking (Leidl et al., 2014; Flanagan et al., 2020). There is also a lack of research on further integrating mechanisms of melatonin therapy considering several protective effects, including DNA damage, cell apoptosis, and mitochondrial dysfunction (Doğanlar et al., 2019; Mehrzadi et al., 2020). Furthermore, the causality between melatonin and ocular diseases is still unknown, despite many studies reporting the various melatonin concentrations between patients and control groups; therefore, it needs more prospective clinical studies. Similarly, the clinical trials of melatonin in treating ocular diseases are still insufficient, resulting in the huge obstacles in its transformation. Table 1 lists its representative clinical trials in publications, which can be seen mostly focused on the glaucoma and sleep disorders, while it is rare for other ocular diseases.

**TABLE 1:** Representative clinical trials of melatonin treating ocular diseases.

Type of diseases	Intervention and dosage	Outcomes and measures	Phase and reference
Cataract	Experimental: melatonin 10 mg tablet Other: placebo tablet	Melatonin could provide anxiolytic effects and decrease IOP	Ismail and Mowafi, (2009)
Hypertensive primary open-angle glaucoma	Agomelatine 25 mg/day, oral, 30 days	Agomelatine could decrease approximately 30% IOP	Pescosolido et al. (2015)
—	Daily group: melatonin 1 mg tablet, intake between 22:00 and 23:00, 3 days	Melatonin could reduce the IOP intake after 2 h or daily intake	Carracedo-Rodríguez et al. (2020)
	Acute group: melatonin 1 mg tablet, intake at 11:00, 1 day		
Blindness with non-24 h sleep–wake disorder	2 weeks placebo run-in, 6 weeks randomized placebo or Circadin^®^ 2 mg, 2 weeks placebo run-out	Circadin^®^ improved the sleep difficulties for totally blind individuals	Roth et al. (2015)
Cataract surgery patients with non-exudative AMD	Experimental: yellow Alcon IOL	Primary: Melatonin content of serum during day	Terminated, NCT00444249
	Other: white Alcon IOL	Secondary: Drusen number, retinal thickness, pupil size, sleeping time	
Totally blind	Experimental: 0.5 mg tablet of melatonin, twice a week, 1 year	The efficacy of melatonin treatment in entraining blind free-running children and young adults	Terminated, NCT00795236
	Other: observational		
DR	Experimental: melatonin 3 mg, 8 weeks	Sleep pattern, and melatonin and cortisol rhythm	II, NCT04547439
	Other: placebo, 8 weeks		
DR	Experimental: melatonin 4 mg, 21 day	Primary: Progression of diabetic retinopathy	III, NCT03478306
	Other: placebo, 21 day	Secondary: Pupillary light response after postillumination, circadian photoentrainment and retinal structure	
	Washout 1 week and switching arm		

## Enhancing the Bioavailability

The existing clinical trials about using melatonin to treat ocular diseases are mainly based on oral administration; however, the recent systematic reviews present that its bioavailability was only approximately 15% with significant individual difference owing to the first-pass metabolism in the liver (Harpsøe et al., 2015). The dosage forms of melatonin are also discussed, and the continuous release and absorption dosage forms demonstrate superior efficacy versus immediate release dosage forms. The latter with properties of short half-life and ultrahigh maximal plasma concentrations may further result in low bioavailability due to the deficient absorption and high risk of tolerability issues (Seiden and Shah, 2019). In the current treatment of ophthalmic diseases, the ocular surface is the most common drug delivery route, and the eye drops have been widely used for delivering melatonin in animal experiments; however, their bioavailability should be further examined owing to the ocular barriers (Dal Monte et al., 2020). For bypassing the barriers, the efficacy of intravitreal injection is also examined, especially in treating retinal diseases; however, the previous study reported that the high-dose melatonin injection resulted in degeneration of retinal cells (Yilmaz et al., 2004; Sande et al., 2014; Tao et al., 2020). Therefore, the potential toxicity of melatonin must be further scrutinized for both the ocular surface and retina, and the proper dosages for treatment must be determined, which may be different in various ocular diseases and individuals. Moreover, the appropriate administration time window of melatonin and the therapeutic effect of other administration routes, including subconjunctival injection, should also be further examined based on the large-scale clinical trials.

Novel nanotechnologies provide a promising delivery strategy with high efficiency in penetration into the ocular surface and sustained release, and the nanocarriers for ocular drug delivery are generally divided into four categories according to the geometric structure: 0D-like (D, dimension) nanoparticles, 1D-like nanofibers, 2D-like nanofilms, and 3D-like nanogels (Yu et al., 2020). The melatonin encapsulated by 0D and 1D nanocarriers has been proven to further improve the bioavailability and therapeutic prognosis of ocular diseases (Quinteros et al., 2014; Ahn et al., 2017). Musumeci et al. (2013) found the PLGA-PEG nanoparticles loaded with melatonin synthesized through the solvent displacement method held twice as long as melatonin aqueous solution (8 h *vs.* 4 h) in decreasing intraocular pressure with good tolerability. Cationic and mucoadhesive carriers are the most common melatonin delivery systems for enhancing the ability of permeation across the ocular surface barriers and prolonging their retention time (Hafner et al., 2015; Carbone et al., 2016). Bessone et al. (2020) reported that the melatonin coated by ethylcellulose nanoparticles showed greater penetration into the cornea with slow releasing speed compared with melatonin solution owing to the mucoadhesive effect with mucin, which significantly increased the retinal thickness and reduced approximately 16% apoptosis of retinal ganglion cells in the RD model, indicating the better retinal protective effect. However, the advanced high-dimensional nanocarriers with the characteristics of high drug-loading capacity and stimuli-responsive capacity used in loading melatonin for ocular drug delivery are still rare, which should be further developed and explored. Recently, co-delivery strategies of melatonin have also been proposed based on the synergy effect on therapies, including with glial cell line–derived neurotrophic factor and neuroprotective agents, which demonstrate better prognosis compared with the single drug. The encapsulation of melatonin in multidrug system further enhances the sustained release of formulation; however, the potential adverse effect on pharmacology caused by drug-loading site competition and pharmaceutical cocrystal formation should be considered (García-Caballero et al., 2018; Arranz-Romera et al., 2019). Moreover, the mass production of melatonin nanodrugs is still challenging, and the clinical trials are still lacking.

## Toward the Future

The effect of melatonin in controlling myopia and treating cataract, glaucoma, uveitis and retinal diseases based on the circadian rhythm and its biological activity has been widely discussed, while for ocular surface, the data are insufficient (Crooke et al., 2017; Alkozi et al., 2020). Limited research reported the melatonin could promote the corneal wound healing, improve oxidative stress injuries in the dry eye, and decrease endoplasmic reticulum stress in granular corneal dystrophy type 2 (Choi et al., 2017; Crespo-Moral et al., 2018; Wang et al., 2021a). As the ocular surface might directly get exposed to the eye drops with melatonin, it is necessary for further understanding their potential interactions and influences. Besides, Gil et al. (2019) reported the melatonin and its analogs could promote the tear secretion in terms of volume, indicating that the melatonin could also impact the tear secretion; however, they did not notice the changes of the tear component. Tears are generally rich in proteins and biomarkers, which are helpful in revealing the mechanism in pathogenesis and treatment of diseases; therefore, the tear variations should be further focused before and after treating with melatonin (Chesnokova et al., 2016; Zou et al., 2020). Furthermore, melatonin also demonstrates potential biomarkers for ocular diseases. Many studies have proven that the concentration changes in melatonin could be detected in tears, saliva, or other body fluids during occurrence and development process of certain ocular diseases (Alkozi H. et al., 2017; Kearney et al., 2017; Pontelli et al., 2019). The sensors for melatonin content have also been developed for determination in biological fluids under ultratrace and real conditions (Camargo et al., 2020; Duan et al., 2020; Kumar and Goyal, 2020; Castaldo et al., 2021). Combining such parameter with other physical signs provides favorable application prospects in differential diagnosis and monitoring of ocular conditions, like dry eye, which is difficult to diagnose accurately in clinical work. Therefore, the correlation between melatonin concentration, especially in tears, and different ocular diseases and their stages in large samples based on sensors should be further explored, which might also provide a reference in personalized medication and understanding the role of melatonin in the eye (Teymourian et al., 2020).

Nowadays, stem cells have been widely used in the ocular disease therapy with well-achieved (Salih et al., 2020; Lin et al., 2021). Reprograming endogenous neural stem cells (NSCs) for promoting neuronal regeneration is considered the most promising way to treat retinal diseases and recover visual acuity (Madelaine and Mourrain, 2017). Melatonin has been proven to facilitate this process. Bai et al. (2016) found 10 μm melatonin could enhance the viability and promote proliferation and reprograming of bovine retinal–derived NSCs *in vitro* through inhibiting the p53-p21–mediated apoptotic pathway and regulating DNA methylation. The proliferation of NSC–induced pluripotent stem cells could also be stimulated through the activation of the ERK 1/2 signaling pathway under melatonin. Similarly, Gao et al. (2019) reported the retinal neural stem cell proliferation and its marker, nestin, increased significantly after using melatonin through melatonin receptor one-mediated in ERK and TGF-β/Smad pathways. However, the role of melatonin in regulating other ocular stem cells, such as corneal epithelial stem cells, and the assessment of their effect in treating ocular diseases in animal models are still unknown. Furthermore, melatonin also exhibits regulating ability for exogenous stem cells such as mesenchymal stem cells (MSCs) in viability, proliferation, differentiation, paracrine, and apoptosis through certain signaling pathways like Wnt and MAPK and acts as antioxidant agents to reduce the oxidative stress–induced apoptosis and enhance activity of stem cells (Ping et al., 2017; Chatterji et al., 2018; Lee et al., 2018; Majidinia et al., 2018; Fan et al., 2020; Giannaccare et al., 2020). Such properties of melatonin have been successfully applied in various disease therapies, including chronic kidney diseases, neurodegenerative diseases, and orthopedic disorders, through pretreatment or combining with scaffold, while its application is rare for ocular diseases (Ramezani et al., 2020; Yan et al., 2020; Yoon et al., 2020). Given the superiority and large potential of melatonin in regulating stem cell therapy in ophthalmology, further exploration of its treating methods, effects, and mechanisms should be emphasized, and the preliminary small-scale clinical trials can also be considered, for example, in limbal stem cell deficiency patients.

The concept of “food is medicine” has been considered an important measure in prevention, management, and treatment of chronic diseases, which was proven by several epidemiological studies and case reports (Downer et al., 2020; Goldenberg et al., 2021; Tribble et al., 2021). The integration of foods with clinical practice will help gain a better prognosis, lower medical expenditure, and more general public health recommendations (Lee et al., 2019). Ocular drugs like latanoprost are usually obtained through chemical synthesis; however, the melatonin can also be detected in various edible animals and plants, which creates possibilities for “food is medicine.” The concentration of melatonin in foods depends heavily on the breed, and much higher melatonin is found in the generative organs of plants like seeds, while for animal foods, eggs and fish present higher contents than other meats (Ma et al., 2019). Several studies have observed the increase in the content of melatonin in serum and its metabolite 6-sulfatoxymelatonin in urine after consumption of beer, grape juice, pineapple, orange, and banana (Maldonado et al., 2009; González-Flores et al., 2012; Sae-Teaw et al., 2013). However, certain research reported the increasing amount of melatonin in the body could not match its intake from foods, and the clinical effects of dietary melatonin were still controversial, which might be ascribed to the individual variation, lack of effective detectable biomarkers *in vivo*, and uniform analyzing methods for melatonin in foods (Kennaway, 2017; Meng et al., 2017). Nonetheless, considering the limitation that clinical trials use melatonin in treating ocular diseases in the current situation and the potential synergistic effect with other medicines, the dietary melatonin should be given priority to patients when ingesting compared with synthesized melatonin from perspectives of safety and relative comprehensive nutrition. In this context, the prophylaxis usage of dietary melatonin can also be considered, and the clinical trials should also be facilitated to provide evidence-based interventions with the participation of clinicians and dietitians.

## Data Availability

The original contributions presented in the study are included in the article/Supplementary Material; further inquiries can be directed to the corresponding author.
