# Supplementary material for: Therapeutic Effects of Melatonin on Ocular Diseases: Knowledge Map and Perspective
Source: Front Pharmacol. 2021 Nov 2;12:721869. doi: 10.3389/fphar.2021.721869 (PMC8593251; doi:10.3389/fphar.2021.721869)
Supplement: Supplementary file 1 [file DataSheet1.docx]

Supplementary Material

# Data collection

All the data were extracted from Web of Science (Wos) core collection from Clarivate Analytics, which have been widely used in bibliometric analysis with the advantages of standardized, high quality, comprehensive and multidisciplinary(AlRyalat et al., 2019; Jappe, 2020; Yao et al., 2020). Article, editorial material, letter and review were considered as the main document types in Wos core collection， therefore the search strategies were set as follows: TS= (melatonin) AND SU= (ophthalmology) AND DT= (Article OR Editorial Material OR Letter OR Review)(Berhidi et al., 2010). The searching and collecting was conducted in June 2021, and a total of 339 articles were obtained.

# Bibliometric analysis

All the data were imported into CiteSpace 5.7.R5 for further bibliometric analysis. CiteSpace allows analysis the cutting-edge areas and hotspots of specific research topic through extracting visualizing terms from titles, keywords and abstracts in data based on co-occurrence frequency matrix(Chen, 2006; Liu et al., 2019). On this basis, the cluster of research topics were determined by log likelihood ratio algorithm through assessing the relationship among terms. Modularity Q and weighted mean silhouette S value are the crucial metric in evaluating the properties of the clusters network, and the high value (close to 1) indicating the cluster is of high quality, while >0.2 is acceptable(Özen Çınar, 2020; Waqas et al., 2020).

The burst analysis reveals certain keywords appearing frequently in scientific outputs in a certain period (from begin to end), indicating the edge-leading topics, which can be evaluated by burst strength(Zhu et al., 2021). The lines display the cycles of research in the field, and red bars represent the duration of the burst while the green line segment indicating the keywords were infrequently in scientific outputs.

# References

AlRyalat, S.A.S., Malkawi, L.W., and Momani, S.M. (2019). Comparing Bibliometric Analysis Using PubMed, Scopus, and Web of Science Databases. *JoVE* (152)**,** e58494. doi: doi:10.3791/58494.

Berhidi, A., Csajbók, E., and Vasas, L. (2010). Author-overdose. When will we come clean? – asks the medical librarian. *Orvosi Hetilap* 151(5)**,** 184-192. doi: 10.1556/oh.2010.28761.

Chen, C. (2006). CiteSpace II: Detecting and visualizing emerging trends and transient patterns in scientific literature. *Journal of the American Society for Information Science and Technology* 57(3)**,** 359-377. doi: 10.1002/asi.20317.

Jappe, A. (2020). Professional standards in bibliometric research evaluation? A meta-evaluation of European assessment practice 2005-2019. *Plos One* 15(4). doi: 10.1371/journal.pone.0231735.

Liu, S., Sun, Y.P., Gao, X.L., and Sui, Y. (2019). Knowledge domain and emerging trends in Alzheimer's disease: A scientometric review based on CiteSpace analysis. *Neural Regeneration Research* 14(9)**,** 1643-1650. doi: 10.4103/1673-5374.255995.

Özen Çınar, İ. (2020). Bibliometric analysis of breast cancer research in the period 2009–2018. *International Journal of Nursing Practice* 26(3). doi: 10.1111/ijn.12845.

Waqas, A., Teoh, S.H., Lapão, L.V., Messina, L.A., and Correia, J.C. (2020). Harnessing telemedicine for the provision of health care: Bibliometric and scientometric analysis. *Journal of Medical Internet Research* 22(10). doi: 10.2196/18835.

Yao, L., Hui, L., Yang, Z., Chen, X., and Xiao, A. (2020). Freshwater microplastics pollution: Detecting and visualizing emerging trends based on Citespace II. *Chemosphere* 245**,** 125627. doi: <https://doi.org/10.1016/j.chemosphere.2019.125627>.

Zhu, X., Hu, J., Deng, S., Tan, Y., Qiu, C., Zhang, M., et al. (2021). Bibliometric and Visual Analysis of Research on the Links Between the Gut Microbiota and Depression From 1999 to 2019. *Frontiers in Psychiatry* 11. doi: 10.3389/fpsyt.2020.587670.
